# Genome Plasticity of *agr*-Defective Staphylococcus aureus during Clinical Infection

**DOI:** 10.1128/IAI.00331-18

**Published:** 2018-09-21

**Authors:** Deena R. Altman, Mitchell J. Sullivan, Kieran I. Chacko, Divya Balasubramanian, Theodore R. Pak, William E. Sause, Krishan Kumar, Robert Sebra, Gintaras Deikus, Oliver Attie, Hannah Rose, Martha Lewis, Yi Fulmer, Ali Bashir, Andrew Kasarskis, Eric E. Schadt, Anthony R. Richardson, Victor J. Torres, Bo Shopsin, Harm van Bakel

**Affiliations:** a Department of Medicine, Division of Infectious Diseases, Icahn School of Medicine at Mount Sinai, New York City, New York, USA; b Department of Genetics and Genomic Sciences, Icahn School of Medicine at Mount Sinai, New York City, New York, USA; c Icahn Institute for Genomics and Multiscale Biology, Icahn School of Medicine at Mount Sinai, New York City, New York, USA; d Department of Microbiology, New York University School of Medicine, New York, New York, USA; e Department of Medicine, Division of Infectious Diseases, New York University School of Medicine, New York, New York, USA; f Department of Microbiology and Molecular Genetics, University of Pittsburgh, Pittsburgh, Pennsylvania, USA; University of Illinois at Chicago

**Keywords:** Staphylococcus aureus, gene regulation, genome analysis

## Abstract

Therapy for bacteremia caused by Staphylococcus aureus is often ineffective, even when treatment conditions are optimal according to experimental protocols. Adapted subclones, such as those bearing mutations that attenuate *agr*-mediated virulence activation, are associated with persistent infection and patient mortality. To identify additional alterations in *agr*-defective mutants, we sequenced and assembled the complete genomes of clone pairs from colonizing and infected sites of several patients in whom S. aureus demonstrated a within-host loss of *agr* function. We report that events associated with *agr* inactivation result in *agr*-defective blood and nares strain pairs that are enriched in mutations compared to pairs from wild-type controls. The random distribution of mutations between colonizing and infecting strains from the same patient, and between strains from different patients, suggests that much of the genetic complexity of *agr*-defective strains results from prolonged infection or therapy-induced stress. However, in one of the *agr*-defective infecting strains, multiple genetic changes resulted in increased virulence in a murine model of bloodstream infection, bypassing the mutation of *agr* and raising the possibility that some changes were selected. Expression profiling correlated the elevated virulence of this *agr*-defective mutant to restored expression of the *agr*-regulated ESAT6-like type VII secretion system, a known virulence factor. Thus, additional mutations outside the *agr* locus can contribute to diversification and adaptation during infection by S. aureus agr mutants associated with poor patient outcomes.

## Abstract (precis)

Therapy for bacteremia caused by Staphylococcus aureus is often ineffective, even when treatment conditions are optimal according to experimental protocols. Adapted subclones, such as those bearing mutations that attenuate *agr*-mediated virulence activation, are associated with persistent infection and patient mortality.

## INTRODUCTION

In contrast to organisms that acquire genes for pathogenesis, Staphylococcus aureus appears to have adapted to infection and the hospital environment through virulence-attenuating mutations that partially or completely inactivate the quorum-sensing virulence regulator *agr* (1–8). *In vitro*, the Agr quorum-sensing system coordinates a switch from an establishment mode, in which genes for adhesins and protective surface proteins are expressed, to an invasive mode, in which genes for factors that promote host cell and tissue destruction are activated (reviewed in reference 9). *agr*-deficient mutants are attenuated for virulence in animal models of acute infection, and agents that block *agr* function and quorum sensing exhibit anti-infective properties in animals (10). However, *agr*-defective clinical isolates that are “locked” in a low-cell-density (noninvasive) regulatory state arise during infection, particularly in patients with endocarditis, osteomyelitis, and bacteremia (5, 11–13). In this situation, the *agr*-defective mutants are often associated with persistent infection, the emergence of host and synthetic antimicrobial resistance, and poor outcomes, opposite what is expected from animal infection studies and from the *in vitro* cytotoxic properties of these strains (11, 13–18). Thus, the emergence of naturally occurring *agr*-defective mutants during infection provides an opportunity to study how the pathogen shifts to a more persistent state. Moreover, understanding how S. aureus adapts to the challenges of invasive infection is central to managing serious, complicated disease.

In the present work, we mapped all genetic changes outside the *agr* locus that accompany *agr* mutation in the human host. We examined naturally occurring *agr*-defective mutants from a previously characterized collection of 158 pairs of predominantly methicillin-susceptible S. aureus (MSSA) clones from nasal carriage and from infecting sites of the same patient (12, 19). Strain pairs from individual patients were genotypically isogenic based on pulsed-field gel electrophoresis (PFGE) banding patterns and *spa* types (12, 19). Although *agr*-defective mutants were infrequent in the original study population (5% [19]), among the 158 bacteremic patients, 15 exhibited *agr*-defective S. aureus in blood samples. Of these patients, 5 were nasally colonized with a genotypically isogenic *agr*-positive (*agr^+^*) strain, indicating that a within-host loss of *agr* function had occurred. Moreover, these pairs provided a unique set of strains for comparisons that would allow the identification of mutational changes located outside the *agr* regulon. Complete genome sequencing was used to compare the 5 pairs of clones from nasal and infecting sites that demonstrated a within-host loss in *agr* function and uniformly *agr^+^* clone pairs from patients without a loss.

## RESULTS

### Whole-genome sequencing confirms the relatedness of colonizing and infecting isolates within patients.

Individual clones from all 5 pairs of isolates with a loss of *agr* function in the infecting isolate were examined by whole-genome sequencing (Table 1) (these samples were called cases). Because genetic heterogeneity may not be limited to *agr*-defective strains, we sequenced pairs of clonal isolates from 7 additional subjects as controls. These control isolates did not demonstrate a within-host loss of *agr* function, but they were represented by a bacteremia sample and a colonization sample. To avoid potentially confounding effects of mutations associated with the adaptation of hospital-acquired methicillin-resistant S. aureus (MRSA) strains to the health care environment, which in some cases may have occurred decades ago (1, 3), we examined both MSSA and MRSA (see Table S1 in the supplemental material), since unlike MRSA, MSSA clones usually do not disseminate in hospitals (20, 21). Thus, whole-genome sequencing was expected to provide a broad characterization of the range and potential diversity of mutations associated with human bacteremia. Case and control groups were balanced for MSSA/MRSA, each containing two patients with MRSA isolate pairs as determined by the presence of *mecA*. In an additional 3 instances (2 cases and 1 control), available isolates from the presumed focus of infection (e.g., pneumonia or skin and soft tissue infection) were included in the analysis to assess their roles as reservoirs for variants.

**TABLE 1:** Summary of variants in infecting strains compared to the colonizing strain in each patient

Patient	Strain type	*agr* status^*a*^	No. of SNVs						No. of insertions/deletions of ≤5 nt		No. of structural variants of >5 nt					Total no. of variants^*d*^ (no. of variants in core genome/no. of variants in accessory genome)
			Nonsynonymous (I/C/U)^*e*^	Stop gain (I/C)^*e*^	Stop loss (I/C)^*e*^	Synonymous (I/C/U)^*e*^	Intergenic (I/C/U)^*e*^	Other^*b*^	Intergenic (I/C)^*e*^	Frameshift (I/C)^*e*^	Structural variant (I/C)^*e*^	Repeat expansion (I/C)^*e*^	Variable region^*c*^	Inversion	Plasmid loss	
Cases																
53	Blood	−	46/48/7	1/1	0/0	21/49/37	21/20/52	7	13/13	5/6	3/3	3/4	1	0	1	362 (137/225)
60	Blood	−	0/1/0	0/0	0/1	0/0/0	0/0/1	1	0/0	0/1	0/0	0/0	0	0	0	5 (4/1)
73	Blood	−	5/6/0	0/0	0/0	4/3/37	4/1/0	0	1/2	1/0	2/0	1/1	0	0	0	68 (22/46)
117	Blood	−	0/0/2	0/0	0/0	0/1/0	2/0/0	0	1/0	0/0	0/2	0/0	0	0	0	8 (4/4)
117	Focus	+	1/0/0	0/0	0/0	1/1/0	1/0/0	0	0/1	0/0	0/0	0/0	0	0	0	5 (3/2)
135	Blood	−	1/3/2	0/0	0/0	0/1/1	0/3/1	0	3/4	0/1	3/3	0/1	0	0	1	28 (6/22)
135	Focus	−	1/3/2	0/0	0/0	0/1/1	0/3/1	0	6/0	1/1	4/3	0/1	0	0	0	28 (5/23)
Controls																
35	Blood	+	0/2/0	0/0	0/0	0/0/0	0/0/0	0	0/0	0/0	0/0	1/0	0	0	1	4 (0/4)
36	Blood	+	0/2/0	0/0	0/0	0/0/0	0/0/0	1	1/1	0/0	0/0	1/0	0	0	0	6 (3/3)
45	Blood	+	1/0/0	0/0	0/0	0/0/0	0/0/0	0	0/0	0/0	0/0	0/0	0	0	0	1 (1/0)
63	Blood	+	2/2/0	0/0	0/0	0/0/0	0/0/0	0	0/0	0/0	0/0	0/0	0	0	0	4 (4/0)
108	Blood	+	0/0/0	0/0	0/0	0/0/0	0/0/0	0	0/0	2/1	0/0	0/0	0	0	0	3 (2/1)
152	Blood	+	0/0/0	0/0	0/0	0/1/0	0/0/0	0	0/0	0/0	1/1	0/0	0	0	0	3 (2/1)
152	Focus	+	0/0/0	0/0	0/0	0/1/0	0/0/0	0	0/0	0/0	1/2	0/0	1	0	0	5 (1/4)
158	Blood	+	1/1/0	0/0	0/0	0/0/0	0/0/0	0	0/0	0/0	0/0	0/0	0	1	0	3 (2/1)

a +, wild type; −, *agr* defective.

b Uncategorized SNV in the gene in one strain but not in the gene in the other.

c Variable region with a different sequence at the same position.

d Variant counts in the total, core, and accessory genomes.

e Ancestry of mutations was determined by PAML analysis. I, infecting strain (blood or infection focus); C, colonizing strain (nares); U, undetermined ancestry (i.e., associated with variably present repeat and/or mobile elements).

**FIG 1:**
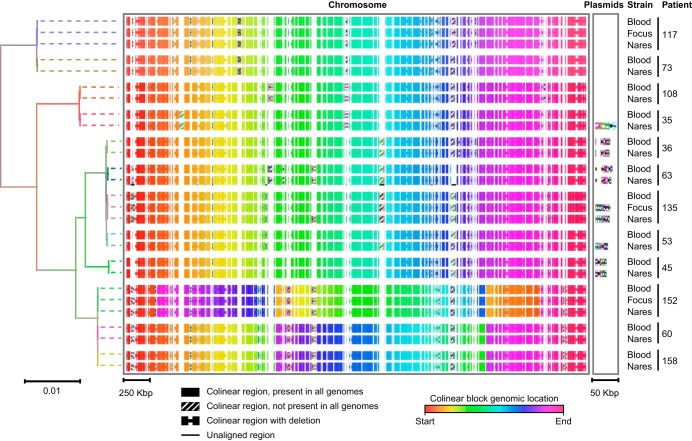
Genomic comparison of study strains with block alignments. Shown is a maximum likelihood phylogenetic tree based on core genome SNVs of all patient clones (left), with a graphical representation of the complete genome alignment (right). Branches in the phylogenetic tree are colored according to the patient from whom each strain originated. Bars indicating the number of substitutions per site in the phylogenetic tree or the alignment block length are shown at the bottom. Dotted lines are included in the tree as guides and do not reflect genetic distance. Core colinear blocks present in all isolate genomes are shown as solid rectangles in the multiple alignment and are colored according to the block location in each genome to highlight inversions (key at the bottom). Noncore regions present in only a subset of genomes are each represented with a unique striped fill pattern.

Closed genomes were obtained by using Pacific Biosciences (PacBio) RS-II long-read sequencing for all 27 isolates (5 pairs of case strains, 7 pairs of control strains, and 3 foci), including 9 distinct plasmids from 6 patients. Additional Illumina sequencing was performed to address insertion/deletion (indel) errors associated with homopolymer regions in the PacBio data, resulting in a correction of 4 to 159 variants per genome. A description of strains, genome size, sequence quality, and the presence of plasmids can be found in Table S1. Phylogenetic analysis and multisequence alignment of the genomic data confirmed that blood, infection focus, and nares strains from each patient were more closely related to each other than to strains from other patients (Fig. 1) or to 39 reference strains having completely sequenced genomes (obtained from the NCBI) (Fig. S1).

We identified mutations in cases and controls by comparing the complete genome of each infecting strain (i.e., blood or infection focus) to that of the colonizing strain (i.e., nares) from the same patient. In aggregate, our analysis identified 420 single nucleotide variants (SNVs), 66 indels of <5 nucleotides (nt), 44 larger structural variants (SVs) (>5 nt), and 3 plasmid losses across all strain pairs, which we further categorized by type and predicted impact on coding sequences (Table 1). Variants were unevenly distributed across genome pairs (Fig. S2), related to whether they were located within core genomic regions present in all sequenced strains and complete NCBI genomes (Fig. S1) or within accessory genomic regions found in only a subset of strains. The number of variants per megabase was 6.3-fold higher in the accessory genome, suggesting a higher rate of evolution than in the core genome. Notably, variants associated with tandem repeat (TR) regions in the accessory genome, which would not be readily detected without long-read sequencing (22), contributed substantially to the number of SNV differences between colonizing and infecting strains of patient 53 (101 SNVs in 5 TR regions) and patient 73 (34 SNVs in one TR region) with a loss of *agr* function. Presumably, the SNVs in these regions do not represent independent events but rather resulted from repeat expansions and contractions.

The largest numbers of core (*n* = 137) and accessory (*n* = 225) variants between the blood and nares strains were observed with patient 53 (this patient acquired *agr*-defective, catheter-associated bacteremia in an adult intensive care unit). The number of variants is large, considering that the reported mutation rate is 2.7 to 3.3 mutations per Mb per year for the S. aureus core genome (23, 24). Nevertheless, at least 2 observations support the idea that the two strains arose from a common ancestor in the same individual host. First, the *agr*-defective infecting and *agr^+^* colonizing isolates shared the same pulsed-field gel electrophoresis (PFGE) and *spa* types (Table S1), which were found in only 1.3% (*n* = 5) of isolates from the two patient populations (158 cases plus 229 uninfected controls) in the parent study (12, 19). Mixed infection with such a rare subtype is unlikely. Second, genomic sequencing analysis for patient 53 indicated that the *agr*-defective infecting isolate was much more closely related to the *agr^+^* colonizing isolate than to all 5 genotypically related strains from the parent study (Fig. S1), arguing against superinfection by a locally circulating clone. Thus, the clonality of isolates from patient 53 was confirmed, consistent with genotyping results from the original studies (12, 19) and other work indicating that carriage is the most common origin of S. aureus infection (8, 19, 25–27).

### Loss of *agr* function is associated with genomic divergence of colonizing and infecting strains.

The number of sequence differences between strain pair genomes from cases with a loss of *agr* function (range, 5 to 362) was significantly higher than for controls (range, 1 to 6) by a nonparametric Wilcoxon test (*P* = 3.25 × 10^−3^), even after excluding focus strains (*P* = 4.67 × 10^−3^) and patient 53 (*P* = 1.01 × 10^−2^) from the analysis. The increased number of variants accompanying the loss of *agr* in the infecting strains was apparent in both the core and accessory genomes and across all variant types. Thus, within-host loss of *agr* function was associated with increased genetic divergence between the colonizing and infecting subclones. We did not find significant differences in mutation frequencies between MRSA and MSSA isolates among cases or controls. The variant pattern of the three focal isolates closely matched that of the blood isolates in most cases (Table 1), consistent with their role as the clinically presumed focus of infection.

To address the directionality of mutation, we reconstructed the ancestral sequence for the set of subclones from each patient using the PAML package (28). Nucleotide sequence variations between strains from blood, foci, or nares were compared to the ancestral sequence to infer the mutation ancestry. By performing phylogenetic analyses with closely related reference strains (see Fig. S1 in the supplemental material), we inferred ancestry for 268 of the 420 single nucleotide variants (Table 1 and Fig. S2). The remaining 144 SNVs were located mostly in accessory genome regions; recombination events associated with TRs and mobile genetic elements in these regions preclude reliable reconstruction of their ancestry (24). Likewise, structural variants and indels that are subject to similar constraints were omitted from the PAML analysis; they are discussed below. Analysis of the number of synonymous substitutions per synonymous site (*dS*) (silent) relative to the number of nonsynonymous substitutions per nonsynonymous site (*dN*) (amino acid altering) indicated that variants were overall under negative selection (ratios of 0.58 and 0.32 for blood and nares isolates, respectively). However, *dN/dS* ratios can be difficult to interpret for sequence data of a population in which variants have not yet fixed (29). When considering only mutations specific to the infecting strains, *agr*-defective strains from cases showed an increased mutational burden relative to *agr^+^* infecting strains from controls (*P* = 0.023). Moreover, the cases with a loss of *agr* functionality showed comparable numbers of mutations in colonizing and infecting strains (Table 1). This suggests that the parental wild-type (WT) strains and *agr*-defective variants evolved concurrently, resulting in substantial genetic divergence between the colonizing and infecting isolates within a given patient.

### Variations in *agr*-defective strains impact genes vital to cell processes and virulence.

**FIG 2:**
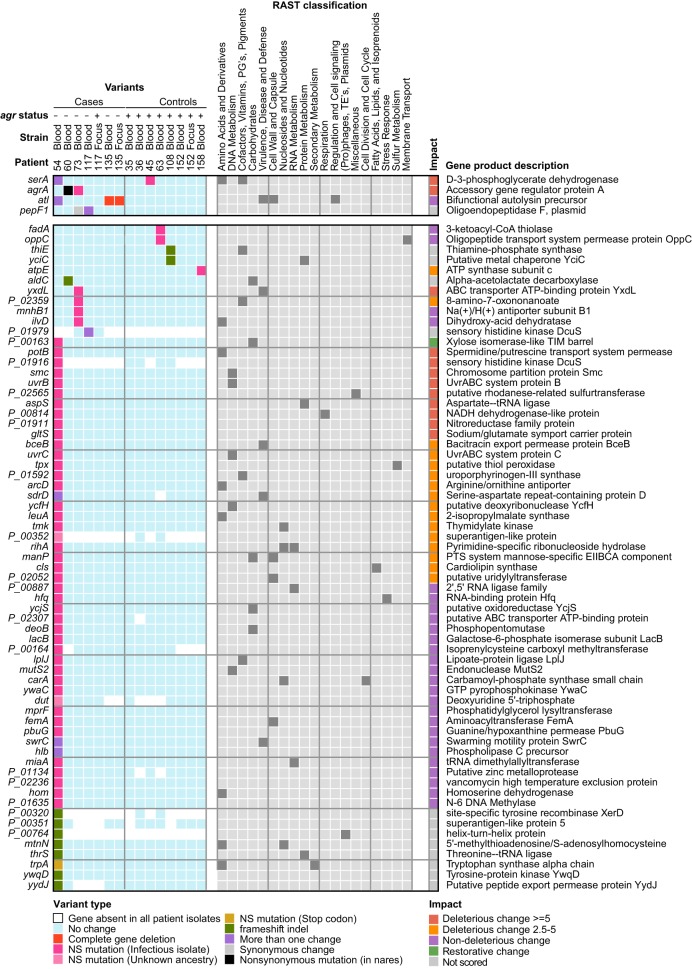
Map of SNVs and indels found in infecting strains. Shown is a mutation matrix of genes (rows) affected by variants of <5 nt that are unique to infecting strains (columns) or for which an ancestral state could not be determined. Genes mutated in multiple patients are grouped at the top, and the color key for different mutation types is shown at the bottom. RAST subsystem classifications associated with each gene are shown in the center (gray). PROVEAN scores (90) were calculated for each nonsynonymous mutation to assess the impact of a variant on the biological function of the encoded protein and are displayed in the rightmost column, with the key shown below. In cases where multiple variants were found in a gene, the PROVEAN score with the highest absolute value for each gene is shown. PG's prosthetic groups; TE's, transposable elements; PTS, phosphotransferase system.

Nonsynonymous SNVs (NS-SNVs) and frameshift indels specific to *agr*-defective blood strains occurred within genes whose products are involved in metabolism, cell wall synthesis, and the DNA repair/damage response, along with several stress response, regulatory, and drug/metal resistance genes (Fig. 2; see also Table S2 in the supplemental material). All predicted amino acid changes were unique to individual strains, suggesting that polymorphisms did not reflect “hot spots” for mutation within particular genes. However, although most genes were mutated in only one of the strains, three (*serA*, *atl*, and *pepF1*) were independently mutated in different patients (Fig. 2), in addition to the recurrent *agrA* mutations identified previously (12). For example, two unique NS-SNVs in *serA*, which encodes an enzyme involved in serine biosynthesis, were identified in *agr*-defective blood strains of patients 45 and 53. One gene, *malL*, encoding an oligo-1,6-glucosidase, was recurrently mutated in the *agr^+^* nares strains of two patients (patients 73 and 158). Parallelism of mutated genes in different strains suggests within-host adaptation.

The *agr*-defective blood strain of patient 53 had a multitude of genetic changes, including genes having multiple mutations and mutations embedded in the same pathways. For example, *swrC* was altered by an NS-SNV and by an insertion. Likewise, the *cflB* gene was altered by an insertion, an NS-SNV, and a synonymous mutation. Multiple mutations would be expected if genes are not transcribed and their sequences are not subjected to selection. However, some genes harbored primary and secondary mutations that are predicted to be deleterious, which may signal genetic instability that can enhance adaptation under certain conditions, such as when selection favors a phenotype requiring a combination of two or more mutations that are individually neutral or deleterious (30).

The highly mutated blood strain from patient 53 harbored an NS-SNV in *uvrABC*, which is a potential mutator gene that is induced by DNA damage as part of the SOS regulon (31, 32). In order to assess whether the *uvrABC* mutation could have contributed to the extensive genetic differences between the blood and nares strains, we assessed the frequency of mutation to rifampin resistance in patient 53. In contrast to the comparison of control isogenic WT and *mutS*-inactivated hypermutator strains, the *agr*-defective strain and its parent strain showed similar mutation frequencies *in vitro*, ∼1 mutation per 10^7^ cells (Fig. S3), indicating that the genetic diversification between the blood and nares strains from patient 53 was unrelated to a *uvrABC* mutation.

### Structural variants in *agr*-defective strains.

Our high-quality complete genome assemblies enabled the identification of structural variants and plasmid losses that distinguished blood from nares strains (Table 2; see also Table S3 in the supplemental material). Structural variants consisted of deletions of prophage and insertion sequences, tandem repeat contractions and expansions, and indel events of >5 bp. The ancestral state for indels and SVs could not be determined by using sequence alignments; however, SVs were more common (28 versus 8) among patients with a loss of *agr* function (Table 2). Moreover, phage-associated gene loss was more frequently observed in *agr*-defective infecting strains. For example, nares and blood strains of patient 135 differed by a 43,757-bp prophage similar to Staphylococcus phage SA13, which contains a variety of genes, including *atl* and *dnaC* (DNA helicase). An NS-SNV in *atl* and a deletion of *dnaC* were identified in the *agr*-defective variant from this patient, suggesting parallel evolution. Additionally, prophage φSa3 was excised in the *agr*-defective blood strain of patient 53. φSa3 excision occurs frequently in S. aureus, particularly during infection and antimicrobial treatment (33–35). φSa3 inserts into the beta-toxin gene, inactivating the gene in the majority of S. aureus clonal groups (36, 37). Excision of φSa3 restores beta-toxin production but at the same time results in the loss of φSa3-borne virulence factors, such as *sak* and *scn*. As such, φSa3 mobilization is thought to alter virulence properties of the strains.

**TABLE 2:** Structural variants and plasmid losses in infecting compared to colonizing strains in each patient

Patient	Strain type	*agr* status^*a*^	Type of event	Fragment length (bp)	No. of genes	Event description
Cases						
53	Blood	−	Insertion	4,156	7	Insertion of 4 paralogs of *essI* and 4 genes encoding hypothetical proteins in the *ess* locus
			Repeat expansion	120	1	Expansion in gene *sdrD*
			Repeat contraction	90	1	Contraction in gene *sdrE*
			Deletion	21	0	Deletion in intergenic region
			Repeat contraction	69	0	350 bp upstream of *setC*
			Repeat expansion	57	0	Intergenic region
			Repeat contraction	56	0	Intergenic region
			Deletion	1,674	0	Deletion of 4 genes encoding hypothetical proteins and a partial deletion of 1 gene encoding a hypothetical protein
			Repeat expansion	59	0	Intergenic region
			Deletion	43,046	66	Deletion of a φSa3 prophage containing *scn*, *sak*, *lytN*, *dnaC*, *xerC*, and 61 genes encoding hypothetical proteins; excision restores *hlb*
			Repeat contraction	100	0	Intergenic region
			Repeat expansion	6	1	Expansion in *swrC*
			Insertion	1,152	0	Intergenic region
			Repeat expansion	128	1	Expansion in *clfB*
			Plasmid loss	23,160	24	Loss of plasmid containing *racA*, *entD*, *entA*, *entG*, *acuI*, *ohrR*, bin3, *cadC*, and 16 genes encoding hypothetical proteins
60	Blood	−	None			
73	Blood	−	Repeat expansion	90	1	Expansion in gene *bbp*
			Repeat contraction	23	1	Expansion in a gene encoding a hypothetical protein
			Insertion	1,074	1	Insertion of a gene encoding a hypothetical protein
			Insertion	60	0	Insertion in the intergenic region
117	Focus	+	None			
117	Blood	−	Deletion	27	1	In gene *pepF*
			Deletion	30	1	In a gene encoding a hypothetical protein
135	Focus	−	Insertion	462	1	Insertion alters a gene encoding a hypothetical protein
			Deletion	462	1	Deletion of a gene encoding a hypothetical protein
			Deletion	43,757	69	Prophage deletion; contains *atl*, *dnaC*, *hin*, *lytN*, *pezA*, and *ssb*
			Insertion	20	0	Insertion of 20 bp in the intergenic region
			Deletion	125	1	Deletion alters a gene encoding a hypothetical protein
			Repeat contraction	22	1	In a gene encoding a hypothetical protein
			Insertion	1,332	2	Insertion in *dpiB*; contains a gene encoding a hypothetical protein
			Insertion	1,332	24	Insertion of a gene encoding a hypothetical protein into plasmid pPS00077.1A.1
135	Blood	−	Insertion	462	1	Insertion alters a gene encoding a hypothetical protein
			Deletion	462	1	Deletion of a gene encoding a hypothetical protein
			Deletion	43,757	69	Prophage deletion; contains *atl*, *dnaC*, *hin*, *lytN*, *pezA*, and *ssb*
			Insertion	20	0	Insertion of 20 bp in the intergenic region
			Deletion	125	1	Deletion alters a gene encoding a hypothetical protein
			Repeat contraction	22	1	In a gene encoding a hypothetical protein
			Insertion	1,332	2	Insertion in *dpiB* contains a gene encoding a hypothetical protein
			Plasmid loss	26,241	23	Loss of a plasmid containing *nhaX*, *dauA*, *hin*, *bin*, *tnsB*, *blaI*, *blaR1*, *blaZ*, *repB*, *qacC*, *norG*, and 10 genes encoding hypothetical proteins
Controls						
35	Blood	+	Repeat expansion	56	0	238 bp upstream of the *hslO* gene
			Plasmid loss	39,353	41	Loss of a plasmid containing *etb*, *hin*, *yxlF*, *ccr*, *lagD*, *ltnA2*, *cadC*, and 35 genes encoding hypothetical proteins
36	Blood	+	Repeat expansion	1,548	3	Alters a gene encoding a hypothetical protein
45	Blood	+	None			
63	Blood	+	None			
108	Blood		None			
152	Focus	+	Insertion	1,332	2	Deletion of gene encoding hypothetical protein alters another gene encoding a hypothetical protein
			Deletion	1,332	1	Insertion alters a gene encoding a hypothetical protein
			Variable region	578/1,330		Deletion of a gene encoding a hypothetical protein; the inserted sequence alters another gene encoding a hypothetical protein
			Deletion	581		Partial deletion of the *mpr* gene
152	Blood	+	Insertion	1,332	1	Contains a gene encoding a hypothetical protein
			Deletion	1,332	2	Deletion of a gene encoding a hypothetical protein; alters another gene encoding a hypothetical protein
158	Blood	+	Inversion	6,939	1	Contains *aphA*, *aadK*, *ycgJ*, and 6 genes encoding hypothetical proteins

a +, functional; −, nonfunctional.

In 2 control patients and 1 case patient, a plasmid present in colonizing strains was absent from infecting strains. Thus, plasmids followed the pattern of prophage-associated gene loss during infection. The 3 deleted plasmids contained a variety of genes involved in the stress response, virulence, and resistance to antimicrobials and metals (Table 2).

### Naturally occurring non-*agr*-associated mutations ameliorate *agr*-defective phenotypes.

To obtain a more detailed assessment of how mutation remodels *agr*-defective mutants, we screened for phenotypes in isolates from patient 53 after controlling for the status of the *agr* regulon. We focused our analyses on this strain set, reasoning that some of the complex mutations seen in the blood strain could compensate for the requirement for *agr* for producing well-known phenotypes *in vitro* and *in vivo*. We engineered an *agr* knockout of the wild-type nares strain (Δ*agr*) and complemented the naturally occurring *agr* mutant blood strain (Δ*agr*::*agr-I^+^*) (see Fig. S4 in the supplemental material). Wild-type *agr* genes were transduced in single copies to the naturally occurring *agr*-defective variant by using the staphylococcal pathogenicity island 1 (SaPI1) *att_C_* locus as the insertion site, as described previously (38).

**FIG 3:**
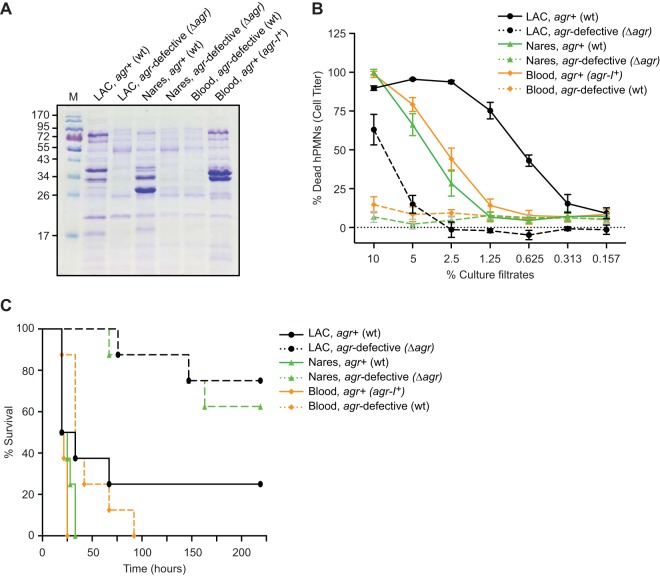
Phenotypic characterization of clinical and genetically manipulated strains from patient 53. (A) Exoprotein profiles of strains grown in TSB for 5 h. Extracts were prepared from culture supernatants and analyzed by sodium dodecyl sulfate-polyacrylamide gel electrophoresis and Coomassie blue staining. M, protein ladder. (B) Intoxication of primary human neutrophils (hPMNs) with culture filtrates from the indicated S. aureus strains and controls (USA300 LAC wild type and *agr* mutant) as a percentage (vol/vol). Results represent the standard errors of the means (SEM) of data from 5 donors and 2 independent colonies. (C) Survival among mice infected with the indicated strains via intravenous inoculation (1 × 10^8^ CFU). Mouse survival results are for 15 mice per group. *P* values for differences in survival were determined by Bonferroni-corrected log rank (Mantel-Cox) tests (*P* = 1.03 × 10^−1^ for *agr*-defective LAC versus *agr*^+^ LAC, *P* = 1.20 × 10^−3^ for the *agr*-defective nares strain versus the *agr*^+^ nares strain, *P* = 1.92 × 10^−2^ for the *agr*-defective blood strain versus the *agr*^+^ blood strain, *P* = 1 for the *agr*-defective nares strain versus *agr*-defective LAC, *P* = 1.80 × 10^−3^ for the *agr*-defective blood strain versus *agr*-defective LAC, and *P* = 2.40 × 10^−3^ for the *agr*-defective blood strain versus the *agr*-defective nares strain).

We next characterized the naturally occurring and engineered *agr^+^* and *agr*-defective variants of the infecting strain from patient 53 and its colonizing counterpart for *in vitro* virulence properties. The *agr* locus is required for the production of many of the S. aureus secreted virulence factors, as exemplified by S. aureus strain LAC (Fig. 3A) (39). Similarly to the control isogenic strain LAC, the nares and blood *agr*-defective mutants from patient 53 demonstrated nearly identical reduced-exoprotein profiles (Fig. 3A). The protein banding pattern from the complemented *agr^+^* blood infection mutant was subtly different from that of the wild-type *agr^+^* nares strain, indicating that *agr* functionality did not entirely account for differences in relative exoprotein abundances. Cytotoxicity assays of cell extracts indicated that the nares and blood *agr^+^* strains were characterized by high cytotoxicity. In contrast, the naturally occurring *agr*-defective mutant and the *agr* knockout strain showed similarly weak killing of neutrophils (Fig. 3B), consistent with the downregulation of many exoprotein genes by the inactivation of *agr* (39–41). Collectively, these data indicate that non-*agr*-associated mutations in the *agr*-defective strain had minor effects on the exoprotein expression pattern and cytotoxicity beyond those associated with *agr* inactivation.

To determine whether the complex genetic alterations in the *agr*-defective blood strain could modulate virulence *in vivo*, we infected mice systemically with the strain set from patient 53 and monitored survival during this bacteremic infection. In contrast to its attenuated cytotoxicity phenotype *in vitro*, the naturally occurring *agr*-defective blood strain caused significantly greater murine mortality than the *agr*-defective nares knockout strain (*P* = 2.4 × 10^−3^) and the LAC control (*P* = 1.8 × 10^−3^) (Fig. 3C). The integration of *agr* into the chromosomal SaPI1 insertion site (*att_C_*) led to a further increase in virulence, suggesting that additional mutations in the *agr*-defective blood strain were responsible for restoring murine mortality near the level observed for the *agr*^+^ parental strain (Fig. 3C). Altogether, these data suggest that genetic changes that accompany *agr*-inactivating mutations in this strain enhanced virulence through pathways other than those involved in cytotoxicity.

### Gene expression profiling reveals a compensatory mutation that restores *ess* activity.

**FIG 4:**
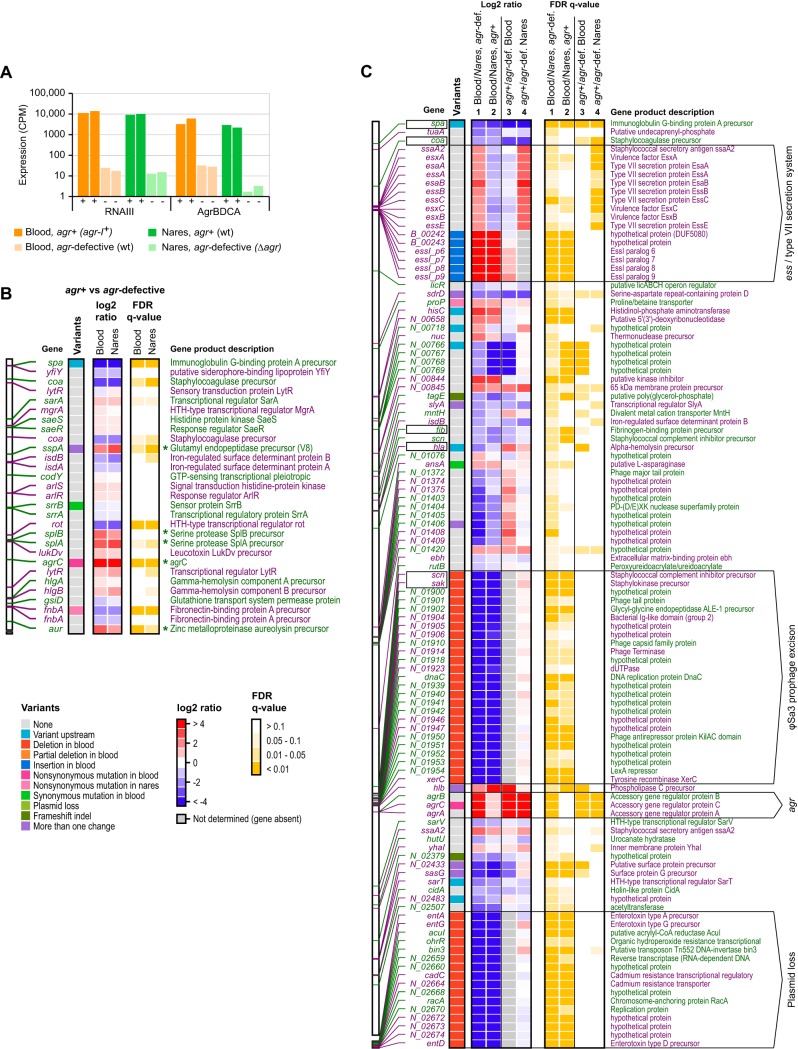
Identification of patient 53 strain-specific changes in gene expression. (A) Bar plot showing expression levels in counts per million (CPM) of RNAIII and *agrBDCA* during late-exponential-phase growth in natural and laboratory-derived *agr*^+^ or *agr*-defective strains of isolates from patient 53. Experiments were performed in duplicate, and levels are plotted for each replicate individually. (B) Overview of expression changes between natural and laboratory-derived *agr*^+^ and *agr*-defective strains of blood and nares origins for 29 toxins, proteases, surface proteins, transporters, and regulatory genes implicated in S. aureus virulence and pathogenesis (42). The left column shows variants found in or near each gene. Center columns indicate the average changes in expression under the experimental conditions labeled at the top. Rightmost columns indicate false discovery rate (FDR)-corrected *P* values for the expression changes shown in the center columns. Identifications and descriptions are shown on the sides, and color keys are shown at the bottom. The position of each gene in the nares strain reference genome is indicated on the far left, and alternating colors of position markers and descriptions are used to denote directly adjacent genes on the same strand (i.e., putative operons). Known *agr*-regulated genes are highlighted by asterisks. Results are derived from the same experiment as the one for panel A. (C) Summary of expression changes for 103 genes with significant differences in expression between *agr*-defective blood (wild-type [wt]) and nares (Δ*agr*) isolates from patient 53. The figure layout is the same as for panel B, and strain comparisons are indicated at the top. Selected genomic regions are annotated on the far right, and virulence genes are boxed on the left. Column numbers and names are shown at the top. HTH, helix-turn-helix; *agr*-def., *agr*-defective.

We next sought to identify signaling pathways that are activated or repressed as a result of the additional mutations found in the *agr*-defective blood strain of patient 53 that could explain its greatly increased virulence in the murine infection model. To this end, we compared transcriptome sequencing (RNA-Seq) expression profiles of the strain set from patient 53 under late-exponential-phase growth conditions. As expected, we observed robust expression of *agr* (Fig. 4A) and induction and repression of known *agr*-regulated genes (42) (for example, protein A, fibronectin binding protein [downregulated], V8 serine protease, and *splA-splB* [upregulated]) in *agr*^+^ compared to *agr*-defective strains (Fig. 4B), thereby indicating *agr* quorum-sensing activity. Some residual expression of *agrBDCA* occurred in the naturally occurring *agr*-defective blood strain compared to the engineered *agr*-defective nares strain (Fig. 4A), likely representing the basal activity of the *agr* P2 promoter, activity seen when the *agr* autoinducing circuit is inactivated owing to a genotypic defect rather than to mobile element replacement (43). Nonetheless, the expression of the RNAIII *agr* effector was reduced by 4 orders of magnitude in both the blood and nares *agr*-defective strains, indicating that *agr* was inactivated.

**FIG 5:**
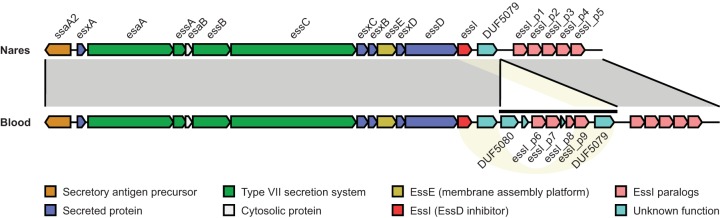
Rearrangement of the *ess* locus in patient 53 blood and nares strains. Matching *ess* regions in the nares (top) and blood (bottom) strains of patient 35 are indicated by shaded areas (gray) and connecting lines. The 4-kb inserted element and candidate regions for homologous recombination are highlighted by a horizontal line and yellow shading, respectively. Gene colors correspond to the type of encoded proteins, according to the key at the bottom.

Overall, the late-log-phase profiles from the *agr*-defective blood and nares strains had similar expression profiles despite their substantial genetic differences (Fig. 4C). In total, 109 genes showed significant (false discovery rate [FDR] *q* value of ≤0.05) expression changes in the naturally occurring *agr*-defective blood strain compared with the engineered *agr*-defective nares strain (33 upregulated and 77 downregulated). Many of the downregulated genes (41/77; 53%) showed a complete loss of expression in the *agr*-defective blood strain due to the loss of prophage φSa3 and a plasmid. For example, the expression of *sak* and *scn* was abolished, and the expression of beta-toxin was increased in the *agr*-defective blood strain, consistent with φSa3 excision (Fig. 4C, column 1). Likewise, we did not observe expression of plasmid-borne virulence factors, such as *ent*, in the *agr*-defective blood strain. Several other virulence factors (*spa*, *coa*, *fib*, and *hla*) were downregulated in both the naturally occurring *agr*-defective and engineered *agr^+^* blood strains compared to their nares counterparts (Fig. 4C, columns 1 and 2). As a notable exception, a gene cluster encoding the *agr*-regulated S. aureus ESAT6-like secretion system (ESS) and ESS-associated virulence factors was significantly upregulated in the *agr*-defective blood strain compared to the engineered *agr*-defective nares strain (Fig. 4C, column 1). Indeed, *ssaA2*, *esaAB*, *essABCE*, and *esxABC* transcripts were restored to levels similar to those seen in the *agr*^+^ colonizing and engineered blood strains (Fig. 4C, columns 2 and 3). The changes in *ess* gene expression were associated with a 4-kb insertion at the locus containing 4 hypothetical proteins and 4 genes encoding paralogs of the EssI inhibitor (Table 2 and Fig. 5). Taken together, the expression changes observed in late log phase, when *agr* is active, suggest partial compensation for the absence of conventional *agr*-mediated gene regulation.

## DISCUSSION

The various stages of invasive infection reflect an array of environmental challenges to S. aureus. Experimental (44–47) and observational (8, 48–51) work suggests that mutation of global regulators constitutes a “one-step” mechanism of adaptation that drives adaptive leaps made by microbes. The present comprehensive identification of variations using complete genome assemblies revealed that the number of mutations in S. aureus clone pairs having a loss of *agr* function is increased compared to uniformly wild-type controls. Although an outlier strain (patient 53) that increased the mean diversity of *agr* mutants existed in the study sample, the increase remains significant even when the outlier is removed from the analysis. S. aureus evolved *in vivo* through the accumulation of point mutations and structural events, such as phage mobilization and plasmid loss.

The increased frequency of non-*agr*-associated mutations that we observed in *agr*-defective strain pairs can be explained by at least two hypotheses. First, systemic synthetic and host antimicrobial exposures in patients could enhance the mutational burden in both colonizing and infecting sites, thereby affecting divergence between the wild-type *agr^+^* strain and the *agr*-defective mutant. Indeed, extensive within-host genetic variation has been described for S. aureus and other pathogens associated with global regulator mutation not only during infection (49, 51–53) but also in the transition between colonization and infection (8, 49, 54, 55). Given that *agr*-defective mutants are associated with long-term invasive infections (15, 18, 56), a second, but not mutually exclusive, hypothesis is that the correlation between *agr* function and the extent of mutations reflects differences in the durations of colonization or infection between cases and controls that we were unable to address in our study. Consistent with this idea, S. aureus isolates obtained from patients with invasive infection demonstrate greater genomic diversity than those obtained from patients with asymptomatic carriage- and those associated with superficial infection (57). In these scenarios, *agr* mutation serves as a proxy for exposure to the necessary milieu and the time required for both the colonizing and infecting bacteria to evolve, potentially resulting in substantial genetic divergence of the two populations.

The frequency and specificity with which compensatory mutations develop in a given patient as a consequence of being infected with an *agr*-defective strain require evaluation of additional strains, virulence, and a detailed examination of clinical histories (e.g., duration of disease and antimicrobials used for treatment). Nevertheless, the number of isolates studied here is sufficient to reveal that additional mutations can create complex changes that may provide a substrate to optimize the within-host specificity of *agr*-defective mutants. Comparative analysis of knockouts and complemented clones from the outlier patient 53 revealed that the *agr*-regulated ESS pathway was highly expressed in the *agr*-defective blood isolate, a finding that was associated with the presence of a 4.1-kb sequence element in genes encoding the *ess* locus inhibitor, EssI. The ESS pathway has been linked to pathogenesis in mouse models of abscess formation (58–60), and it modulates host immune responses, including cytokine production (61). Thus, enhanced expression of *ess* can potentially explain why we observed enhanced virulence of the *agr*-defective strain *in vivo* but not in cytotoxicity assays. Within-host conditions vary, and as a result, the relative benefits of an *ess* mutant phenotype may not apply to individuals other than patient 53. Additionally, other factors, such as φSa3 excision that restored beta-toxin production, may have contributed to the virulent phenotype of the naturally occurring mutant, as beta-toxin is associated with virulence in animal models of infection (35).

Future work will extend our observations on genetic changes in different hosts with *agr*-defective S. aureus infection to investigate diversity within individual hosts. Currently, little is known about intrahost variation in S. aureus genomes during infection, and such uncertainty may be greater in the setting of a high mutational burden. Thus, although the single colony analyzed in this study likely represented the dominant strain in the specimen, further subclonal heterogeneity may have been overlooked.

In conclusion, we find that a loss of *agr* function is associated with increased genomic complexity in colonizing and infecting strains across multiple patients as well as mutations that can potentially compensate for the loss of *agr* function during infection. Such changes may favor the expansion or persistence of S. aureus populations within patients. Nevertheless, by analogy to cancer biology (62, 63), they may also create new vulnerabilities that can be exploited for prognosis and treatment. Recognition of within-host genetic variability associated with global regulator inactivation also has important ramifications for our ability to understand intrapatient and interpatient heterogeneity. Thus, our findings have significance for establishing thresholds to differentiate the relatedness of S. aureus isolates for epidemiological purposes in hospitals.

## MATERIALS AND METHODS

### Bacterial strains and culture conditions.

S. aureus isolates were obtained from a previous multicenter study of bacteremic patients. In the original study, nares cultures were obtained at the time when patients were diagnosed with S. aureus bacteremia (19). The majority of these isolates (92% of blood and 97% of nares isolates) were methicillin-susceptible S. aureus. Isolates were further characterized and screened for *agr* functionality in subsequent work (12). Inactivating mutations in *agr* were identified in most of the ∼10% of blood isolates that were found to be *agr* defective (12).

### Culturing and DNA and RNA extraction.

For genomic DNA (gDNA) extraction, isolates were grown from single colonies in tryptic soy broth (TSB) liquid cultures overnight at 37°C with shaking at 225 rpm. Cells underwent high-molecular-weight (HMW) DNA extraction using enzymatic lysis with lysozyme, lysostaphin, and proteinase K and Qiagen Genomic-Tip columns, as described previously (64).

For RNA extraction, cultures grown overnight were diluted, grown to late log phase (optical density [OD] of ∼0.80), and stabilized in RNAlater. Total RNA was isolated and purified by using the Qiagen RNeasy minikit. Lysozyme and lysostaphin were used for cell wall degradation, followed by two cycles of 2 min of bead beating with 1 ml of 0.1-mm silica beads in a mini-bead beater (BioSpec), and RNA was eluted in nuclease-free water. Isolated RNA was treated with 1 μl of Baseline Zero DNase (Epicentre) at 37°C for 30 min, and rRNA depletion was performed by using an Epicenter Ribo-Zero magnetic gold kit (Illumina), according to the manufacturer's instructions.

### DNA library preparation and sequencing.

Quality control, DNA quantification, and gDNA library preparation and sequencing were performed as described previously (64). Briefly, DNA was gently sheared by using Covaris G-tube spin columns into ∼20,000-bp fragments and end repaired before ligating SMRTbell adapters (Pacific Biosciences). The resulting library was treated with an exonuclease cocktail to remove unligated DNA fragments, followed by two additional (AMPure XP) purification steps and Sage Science Blue Pippin size selection to deplete SMRTbells of <7,000 bp. Libraries were then sequenced by P5 enzyme chemistry on the Pacific Biosciences RS-II platform.

For Illumina sequencing, genomic DNA was sheared to an average fragment size of 200 bp by using a Bioruptor Pico sonicator (Diagenode). Amplicon sequence libraries were prepared by using the end repair, A-tailing, and adapter ligation NEBNext DNA library prep modules for Illumina from New England BioLabs, according to the manufacturer's protocol. Following final purification with AMPure XP beads and secondary PCR (8 cycles) to introduce barcoded primers, multiplexed libraries were sequenced on the Illumina HiSeq 2500 platform in a single-end 100-nt-run format.

### Whole-genome assembly.

PacBio sequencing data were assembled by using the HGAP3 version 2.2.0 assembly pipeline (65), and a custom postassembly pipeline (66) was used to finalize each genome. Briefly, genomes were circularized and reoriented to the origin of replication (*ori*) by using Circlator (67). In cases where chromosomes or plasmids did not assemble into complete circularized contigs, manual curation was performed by using Contiguity (68). Next, Illumina reads were mapped to the curated PacBio assemblies, and consensus calling was performed by using the mpileup function of SAMtools to correct SNVs and small indels in homopolymer regions. To recover small plasmids that might have been lost during size selection of PacBio reads, the Illumina reads were also assembled *de novo* by using SPAdes version 3.7.1 (69). Contigs with <10× coverage and contigs mapping in full to the PacBio assembly were removed. The remaining contigs were circularized by using Circlator or Contiguity and aligned to the nonredundant nucleotide collection by using BLAST^+^ to identify plasmid sequences. Genes were annotated by using PROKKA (70) and visualized by using ChromoZoom (71) and the Integrated Genome Browser (IGB) (72). InterProScan (73) was used to annotate protein domains and gene ontology (GO) categories for annotated genes.

### Phylogenetic analysis.

A set of 80 publically available finished S. aureus genomes listed in the Genomes Online Database (74) were downloaded from RefSeq (http://www.ncbi.nlm.nih.gov/refseq/) and used to select a subset of closely related reference genomes. Briefly, a pairwise comparison of all RefSeq reference strains against all sequenced strains was performed by using MUMmer (version 3.1) (75). MUMi scores (76) were used to calculate the genetic distance between sequenced strains and the RefSeq strains. All RefSeq strains within a short genetic distance from at least one patient isolate (MUMi score of <0.05) were included in the phylogenetic analysis. Parsnp (77) was used to align the subset of RefSeq strains and the sequenced strains, filtering for recombinant regions.

For visualization of the whole-genome alignments, all 23 sequenced strains were aligned by using Mugsy (version 1r2.2) (78). Mugsy alignments were processed with Gblocks (version 0.91b) with default settings and a minimum block size set to 1,000 (79), and a tree was then created by using RAxML (version 8.2.4) (80), using the general time-reversible model. The untrimmed Mugsy alignment was visualized by using ChromatiBlocks (https://github.com/mjsull/chromatiblock).

### Variant calling and ancestral reconstruction.

The ancestral sequence for each set of patient isolates was inferred by using the PAML package (28) (version 1.3.1). Briefly, the phylogenetic tree of all patient strains and 39 complete reference genomes was used to identify a clade of closely related strains for each patient isolate set, within a genetic distance of 0.001 (see Fig. S1 in the supplemental material). We then generated a multiple alignment of the genomes in each clade (78) and used BaseML (from the PAML package) to infer the sequence of the most recent common ancestor for each strain set using a general time-reversible model. As there were no genomes within a genetic distance of 0.001 from the two isolates from patient 45, isolates from patients 135 and 53 (which were closest) were used to infer an ancestral sequence. A custom script (https://github.com/mjsull/GWviz/tree/mssa-paper) was used to determine all SVs from a Nucmer (version 3.1) (75) alignment. The *dN*/*dS* ratios for blood and nares strains were calculated by using yn00, which is part of the PAML software package, using default settings. We also cross-referenced variants with the ancestral sequence to determine whether the variant arose in the nares, infection focus, or blood. A graphic of all variants between these genomes was also generated (Fig. S2). Finally, recombinant DNA was detected within the closely related clades by using Gubbins (version 2.2.0), using default settings (81).

### RAST subsystem assignment.

To determine whether mutations arose in specific classes of genes or pathways, genes were clustered and assigned a subsystem. Genes were matched between patients by their PROKKA-annotated common gene names. If PROKKA did not assign a common name to a predicted gene, genes were grouped if they aligned reciprocally along >90% of their length with an identity of >90% by using BLASTP (82). Subsystems were assigned to each gene annotated with PROKKA by using the RAST annotation server. A list of genes, variants found in each strain, the subsystem assigned to each gene, and the groupings of hypothetical proteins can be found in Table S3 in the supplemental material.

### Directional RNA-Seq.

After RNA extraction, barcoded stranded RNA-Seq libraries were prepared by using the TruSeq stranded total RNA sample preparation kit (Illumina). RNA quality and quantity were assessed by using the Agilent Bioanalyzer and the Qubit RNA broad-range assay kit (Thermo Fisher), respectively. Finally, libraries were pooled and sequenced on the Illumina HiSeq platform in a 100-bp single-end read run format with 6 samples per lane.

### Differential gene expression analysis.

Raw reads were first trimmed by removing Illumina adapter sequences from 3′ ends using cutadapt (83), with a minimum match of 32 bp and allowing for a 15% error rate. Trimmed reads were mapped to the reference genome by using Bowtie2 (84), and htseq-count (85) was used to produce strand-specific transcript count summaries. Read counts were then combined into a numeric matrix and used as the input for differential gene expression analysis with the limma R package (86) in Bioconductor. Normalization factors were computed on the data matrix by using the weighted trimmed mean of M values (TMM) method, followed by voom (87) mean-variance transformation in preparation for limma linear modeling. Data were fitted to a design matrix containing all sample groups, and pairwise comparisons between the groups of interest were performed. eBayes-adjusted *P* values were corrected for multiple testing by using the Benjamini-Hochberg (BH) method and used to select genes with significant expression differences (*q* < 0.05).

### Spontaneous Rif^r^ mutant recovery assay.

Cells were grown in TSB medium for 16 or 48 h at 37°C with constant shaking. Undiluted and serially diluted samples were spotted onto rifampin (5× MIC = 40 ng/ml)-containing tryptic soy agar (TSA) plates and drug-free agar for mutants and total numbers of cells, respectively, as described previously (88). After spot plating, plates were incubated at 37°C, and colonies were counted after 24 h. S. aureus RN6734 *mutS*::pG+host9 (Erm) was generated by transducing the disrupted allele from RN4220 (88). Phage 80α was used to transduce the marker-disrupted allele; transductants were selected on TSA plates containing the appropriate antimicrobial.

### Exoproteomic profiling.

Cultures grown in TSB overnight were diluted 100-fold in 5 ml fresh TSB in 15-ml conical tubes and grown for 5 h at 37°C with constant shaking. The cultures were normalized to the lowest OD at 600 nm (OD_600_) and centrifuged, and the culture filtrates were collected by filtration. Cells were pelleted by centrifugation at 4,000 rpm for 10 min at 4°C, and 1.3 ml of the supernatant was precipitated with 10% trichloroacetic acid (TCA) overnight at 4°C. Proteins were pelleted by centrifugation at 15,000 rpm for 15 min at 4°C, and the pellet was washed with 100% ethanol. The protein pellet was dried and resuspended in 30 μl of TCA-SDS buffer, and 16 μl of the sample was resolved on a 15% SDS-polyacrylamide gel, followed by Coomassie staining.

### Cytotoxicity assays.

Cytotoxicity assays were performed as described previously (89). Briefly, cultures grown overnight in TSB were diluted 100-fold in 5 ml fresh TSB in 15-ml conical tubes and grown for 5 h at 37°C with constant shaking. The cultures were normalized to the lowest OD_600_ and centrifuged, and the culture filtrates were collected by filtration. The supernatants were then mixed with human polymorphonuclear leukocytes (hPMNs) from 6 healthy individuals. Approximately 2 × 10^5^ hPMNs were added to a final volume of 100 μl/well of RPMI (Gibco) supplemented with 10 mM HEPES. Cells were intoxicated for 1 h at 37°C in 5% CO_2_. Ten microliters of CellTiter 96 Aqueous One solution (Promega) was added, the mixture was incubated at 37°C in 5% CO_2_ for 2 h, and the absorbance was measured at 595 nm by using a PerkinElmer EnVision 2103 multilabel reader. Cell survival indicates neutrophil viability in the presence of 0.1% Triton (positive control) and media (negative control).

### Mouse infections.

Five-week-old female ND4 Swiss Webster mice (Harlan Laboratories) were anesthetized intraperitoneally with 250 to 300 μl of Avertin (2,2,2-tribromoethanol dissolved in *tert*-amyl alcohol and diluted to a final concentration of 2.5% [vol/vol] in sterile saline). S. aureus cultures grown for 3 h were washed, resuspended in 1× phosphate-buffered saline, and normalized for the corresponding CFU counts (∼3.5 × 10^7^ to 5 × 10^7^ CFU). One hundred microliters of the inoculum was administered retro-orbitally, and mice were monitored every 4 to 6 h for signs of morbidity (hunched posture, lack of movement, paralysis, and an inability to acquire food or water), at which time the animals were euthanized and survival curves were plotted over time (in hours).

### Accession number(s).

Genome assemblies and RNA-Seq data have been deposited under NCBI BioProject accession no. PRJNA393749.

## Supplementary Material

Supplemental file 1

Supplemental file 2

Supplemental file 3

Supplemental file 4
