# Supplementary material for: Genome Plasticity of agr-Defective Staphylococcus aureus during Clinical Infection
Source: Infect Immun. 2018 Sep 21;86(10):e00331-18. doi: 10.1128/IAI.00331-18 (PMC6204747; doi:10.1128/IAI.00331-18)
Supplement: Supplemental file 1 [file zii999092558s1.pdf]

Supplemental figures for:

Genome plasticity of *agr*-defective *Staphylococcus aureus* during clinical infection

Deena R. Altman*[a,b], Mitchell J. Sullivan*[b], Kieran I. Chacko[b], Divya Balasubramanian[d*], Theodore R. Pak[b], William E. Sause[d], Krishan Kumar[e], Robert Sebra[b,c], Gintaras Deikus[b,c], Oliver Attie[b], Hannah Rose[e], Martha Lewis[b], Yi Fulmer[e], Ali Bashir[b], Andrew Kasarskis[b,c], Eric E. Schadt[b,c], Anthony R. Richardson[f], Victor J. Torres[d], Bo Shopsin[‡d,e], and Harm van Bakel[‡b,c].

a) Department of Medicine, Division of Infectious Diseases, Icahn School of Medicine at Mount Sinai, New York City, NY, USA
b) Department of Genetics and Genomic Sciences, Icahn School of Medicine at Mount Sinai, New York City, NY, USA;
c) Icahn Institute for Genomics and Multiscale Biology, Icahn School of Medicine at Mount Sinai, New York City, NY, USA;
d) Department of Microbiology, New York University School of Medicine, New York, NY, USA ;
e) Department of Medicine, Division of Infectious Diseases, NYU School of Medicine, New York, NY, USA;
f) Department of Microbiology and Molecular Genetics, University of Pittsburgh Pittsburgh, PA, USA

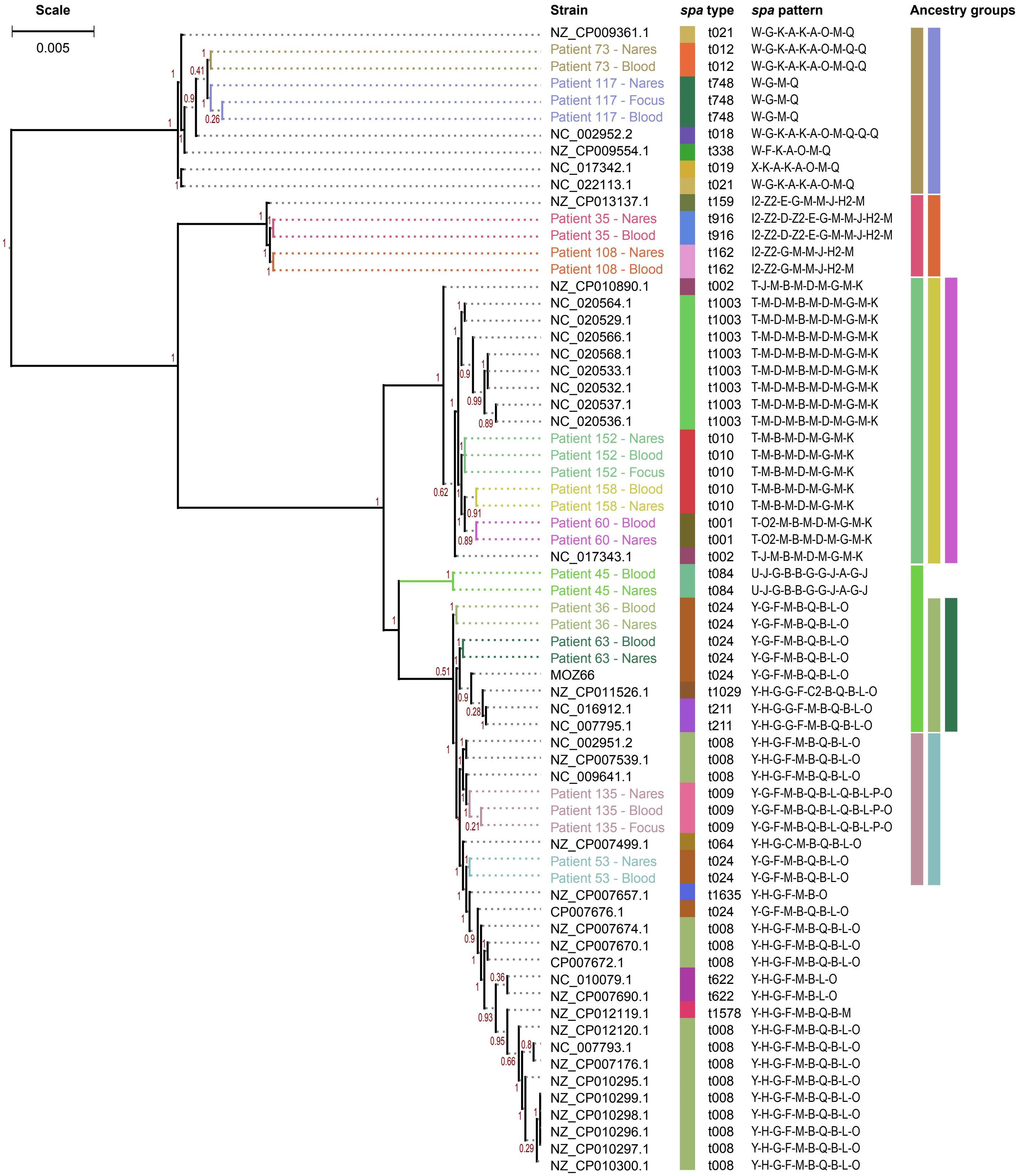

Figure S1. Comparison of patient isolate genomes to other complete *S. aureus* genomes. Maximum likelihood phylogenetic tree based on core genome SNVs of all sequenced patient strain genomes, compared to 39 complete reference genomes obtained from NCBI and the last patient strain from the parent study (MOZ66) that shared the same PFGE-*spa* type as the isolates from patient 53. Matching strain names and colors identify samples from the same patient. Bootstrap values (scaled 0 to 1) are indicated at each node. The selection of genomes used to infer the ancestral state for each set of patient genomes is indicated on the right by color-matched vertical bars. Dotted lines are included in the tree as guides and do not reflect genetic distance.

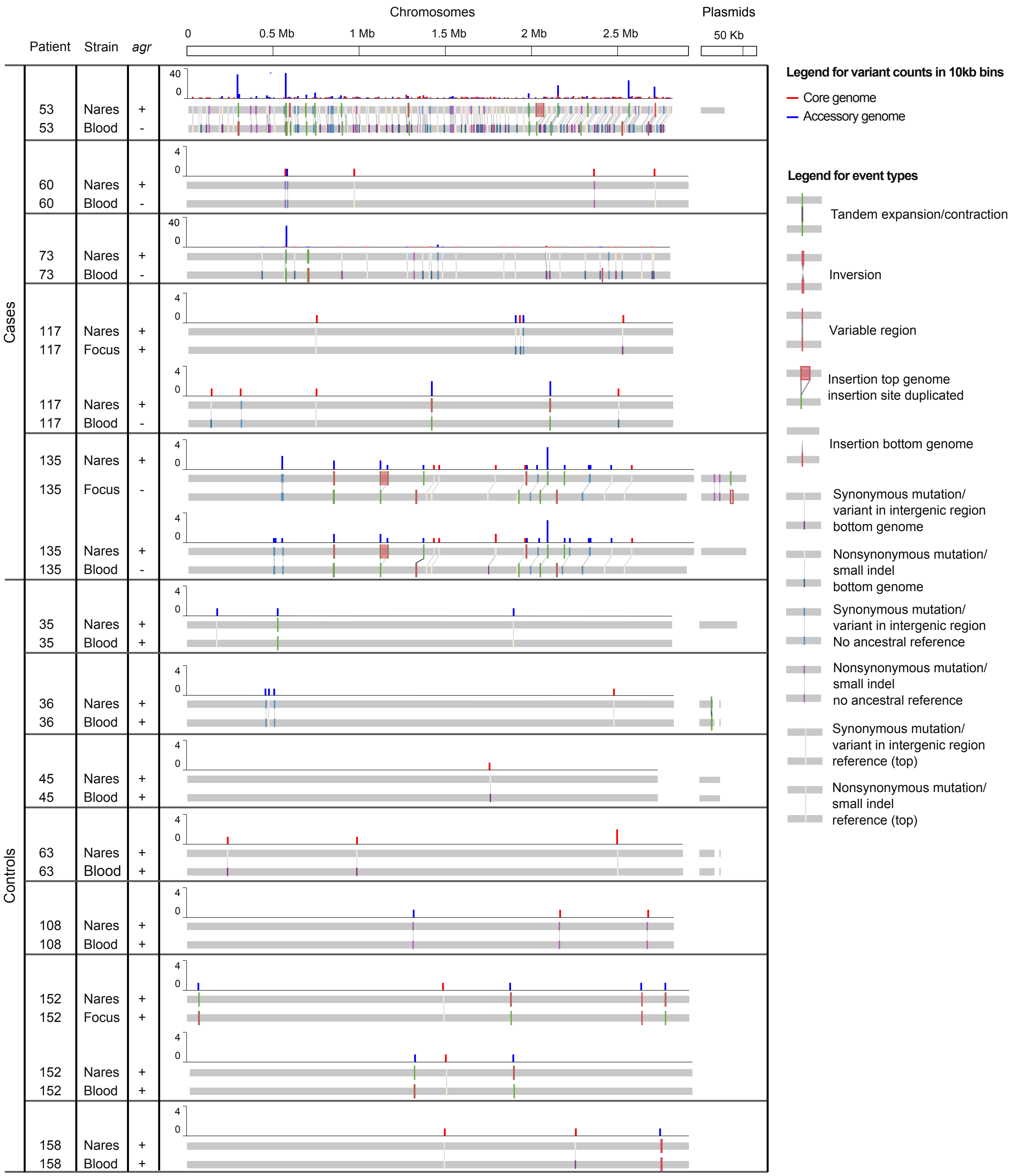

Figure S2. Ancestry assignments for variants identified between genomes from colonizing and infecting patient strains. Pairwise alignments of colonizing (nares) and infecting isolate genomes (blood or infection focus) are shown as sets of horizontal grey bars, with the scale shown at the top, and patient and isolate details shown on the left. For each genome pair, small structural variants (<5nt) and SNVs are indicated as vertical lines with rounded edges, while larger structural variants (>5nt) are indicated as overhanging rectangular blocks. Connecting lines link variant positions in each genome pair. For variants where ancestry could be inferred, darker colors highlight mutations that arose in the infecting isolate while the corresponding ancestral state in the colonizing isolates is shaded lighter. Variants are shaded lighter in both genomes if the mutation occurred in the colonizing isolate, or darker if no ancestry could be inferred. A stacked bar plot with core genome, accessory genome and recombination event-associated variant counts per 10 kb block highlighted in different colors is shown above each pairwise alignment. See legend on the right for further details.

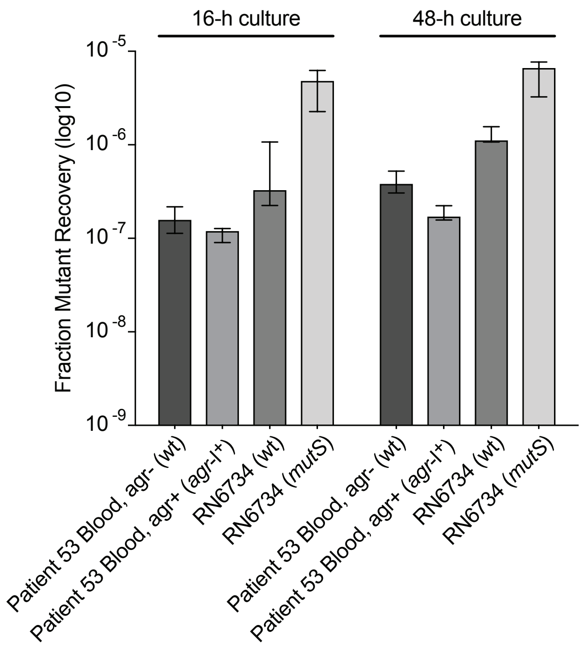

Figure S3. Mutation frequency to rifampicin resistance for colonizing and infecting strains from patient 53. Frequency of spontaneous rifampicin-resistant (RifR) mutants for the indicated *S. aureus* strains and controls. Values are the median and range of three independent measurements. Given that rifampicin-resistant mutants are not selected for during *in vitro* growth, their frequency is an indicator of the overall population mutation rate. Fraction mutant recovery = no. of mutants/total no. of cells. Naturally occurring wild-type (wt) and lab-derived strain genotypes are indicated between brackets for all panels; agr-I = agr-IpJC1111. Wild-type and Δ*mutS* strain RN6734 were used as controls. Inactivation of *mutS*, a key component of the methyl-directed mismatch repair system, increases all major classes of genetic alteration (1).

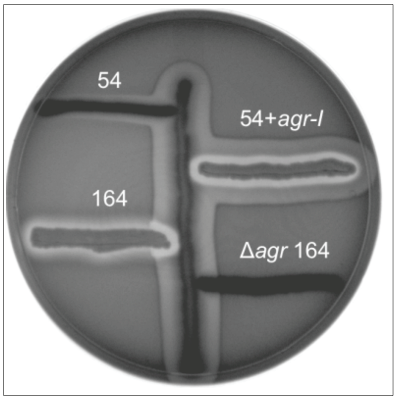

Isolates
164 : Nares
54 : Blood

Figure S4. Cross-streaking of natural and laboratory-derived *agr* knockout and complement strains for patient 53. Strains have been streaked alongside the β-hemolysin producing strain RN4220, which produces only β-hemolysin; differentiation of the various hemolytic activities in *S. aureus* can be scored on sheep blood agar by virtue of their synergism with β-hemolysin (2, 3). Hemolysin production in *S. aureus* can be used to approximate *agr* activity because δ-hemolysin is a translation product of *agr* RNAIII and because α-hemolysin and the phenol-soluble modulins (PSMs; a family of peptides that include δ-hemolysin) are upregulated by RNAIII and *agrA*, respectively (4, 5). Numbers correspond to isolate identifiers as indicated below the figure (see also Table S1).

Supplemental references

1. O'Neill AJ, Chopra I. 2002. Insertional inactivation of mutS in *Staphylococcus aureus* reveals potential for elevated mutation frequencies, although the prevalence of mutators in clinical isolates is low. J Antimicrob Chemother 50:161-9.
2. Cheung GY, Duong AC, Otto M. 2012. Direct and synergistic hemolysis caused by Staphylococcus phenol-soluble modulins: implications for diagnosis and pathogenesis. Microbes Infect 14:380-6.
3. Elek SD, Levy E. 1950. Distribution of haemolysins in pathogenic and non-pathogenic staphylococci. J Pathol Bacteriol 62:541-54.
4. Queck SY, Jameson-Lee M, Villaruz AE, Bach TH, Khan BA, Sturdevant DE, Ricklefs SM, Li M, Otto M. 2008. RNAIII-independent target gene control by the agr quorum-sensing system: insight into the evolution of virulence regulation in Staphylococcus aureus. Mol Cell 32:150-8.
5. Recsei P, Kreiswirth B, O'Reilly M, Schlievert P, Gruss A, Novick RP. 1986. Regulation of exoprotein gene expression in Staphylococcus aureus by agar. Mol Gen Genet 202:58-61.
